# Cell-Free Expression System Derived from a Near-Minimal Synthetic Bacterium

**DOI:** 10.1021/acssynbio.3c00114

**Published:** 2023-06-06

**Authors:** Andrei Sakai, Aafke J. Jonker, Frank H. T. Nelissen, Evan M. Kalb, Bob van Sluijs, Hans A. Heus, Katarzyna P. Adamala, John I. Glass, Wilhelm T. S. Huck

**Affiliations:** † Institute for Molecules and Materials, Radboud University, Nijmegen 6525AJ, The Netherlands; ‡ Department of Genetics, Cell Biology and Development, University of Minnesota, Minneapolis, Minnesota 55455, United States; § Synthetic Biology & Bioenergy, J. Craig Venter Institute, La Jolla, California 92037, United States

**Keywords:** cell-free expression system, *Mycoplasma*, JCVI-syn3A, active machine learning

## Abstract

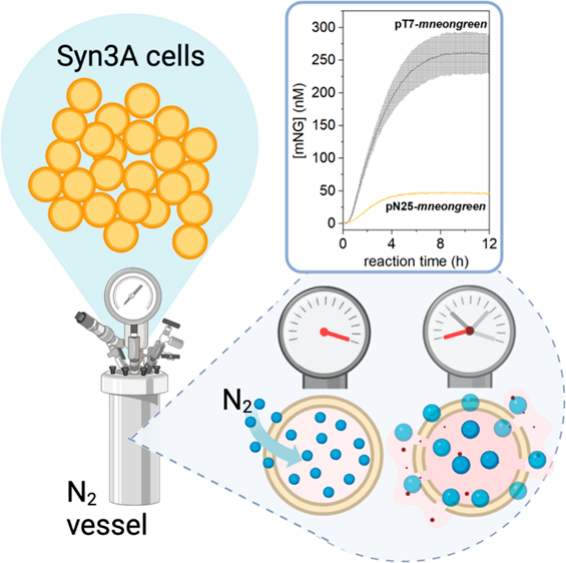

Cell-free expression (CFE) systems are fundamental to reconstituting metabolic pathways *in vitro* toward the construction of a synthetic cell. Although an *Escherichia coli*-based CFE system is well-established, simpler model organisms are necessary to understand the principles behind life-like behavior. Here, we report the successful creation of a CFE system derived from JCVI-syn3A (Syn3A), the minimal synthetic bacterium. Previously, high ribonuclease activity in Syn3A lysates impeded the establishment of functional CFE systems. Now, we describe how an unusual cell lysis method (nitrogen decompression) yielded Syn3A lysates with reduced ribonuclease activity that supported *in vitro* expression. To improve the protein yields in the Syn3A CFE system, we optimized the Syn3A CFE reaction mixture using an active machine learning tool. The optimized reaction mixture improved the CFE 3.2-fold compared to the preoptimized condition. This is the first report of a functional CFE system derived from a minimal synthetic bacterium, enabling further advances in bottom-up synthetic biology.

## Introduction

The bottom-up construction of a living cell is one of the greatest challenges in modern science. For this purpose, the cell-free expression (CFE) system, which enables the *in vitro* expression of proteins from DNA templates, is the basic chassis to build a synthetic cell. One way to achieve this goal is to fill lipid vesicles with the CFE system derived from bacteria and then install a minimal genome that would enliven the assembled parts. This strategy depends on a suitable model organism that can be reconstructed *in vitro* with the currently available technology. *Escherichia coli* (4.6 Mb genome, 4,401 genes) has been widely used as a model organism for the CFE system as it presents vast literature regarding its genetics, structure, and metabolism.^1^ However, *E. coli* is still too complex to be fully reconstructed *in vitro*. *Mycoplasma* bacteria are an example of simpler prokaryotes bearing smaller genomes (0.5–2.2 Mb). The J. Craig Venter Institute minimized the genome of *Mycoplasma mycoides* (1.2 Mb, 985 genes)^2^ into a near-minimal bacterial strain named JCVI-syn3A (0.5 Mb, 493 genes) (Syn3A),^3,4^ making it an attractive model for the bottom-up construction of a synthetic cell.

Another important application of the Syn3A CFE system is to study the minimal cellular metabolism, especially to determine the function of unannotated essential genes present in the Syn3A genome (90 out of 452 protein-coding genes).^4,5^ To date, the elucidation of gene function has relied on metabolic modeling based on experimental information from closely related organisms (e.g., *Mycoplasma mycoides*), transposon experiments, and proteomic data.^4,6^ The Syn3A CFE system can serve as a platform to test the function of a specific gene by reconstructing its immediate metabolic network *in vitro*. Therefore, Syn3A CFE can play an important role in unravelling the function of specific proteins that cannot be explored *in vivo*.

To enable the use of Syn3A for such applications in synthetic biology, a *Mycoplasma*- or Syn3A-derived CFE system seems probably the best CFE platform. Due to the orthogonality between CFE systems derived from distantly related organisms, the *E. coli* CFE system is incapable of expressing *Mycoplasma* genomes. Such orthogonality is mainly caused by differences in codon usage and translational machinery (e.g., aminoacyl-tRNA synthetases).^7^ The expression of bacterial genomes in a heterologous CFE system is rare.^8^ For *Mycoplasma*, only *in vivo* compatibility between closely related *Mollicutes* was observed for genome transplantation into heterologous recipient cell.^9,10^

In previous attempts to derive a functional CFE system from *Mycoplasma* bacteria, we found high ribonuclease (RNase) activity in cell lysates, which impeded *in vitro* protein expression.^11^ We speculated that RNase activity arose from membrane-associated nucleases that were released into lysates during cell disruption. This hypothesis was supported by (i) the degradation of substrate RNA and ribosomes caused by intact *Mycoplasma* cells and (ii) mRNA degradation assays. To reduce the presence of such RNases in lysates, we sought cell disruption methods that allowed a more efficient separation between cytoplasm and membrane.

Here, we report for the first time the production of a functional CFE system derived from Syn3A cells. This was achieved by the production of lysates with considerably lower RNase activity produced using the nitrogen decompression method.^12−14^ Further, we utilized an active machine learning tool to optimize the reaction mixture of the Syn3A CFE system.^15,16^ This enabled us to explore a vast high-dimensional space of reaction mix compositions with minimal experimental work (207 reactions).

## Results and Discussion

**Figure 1:**
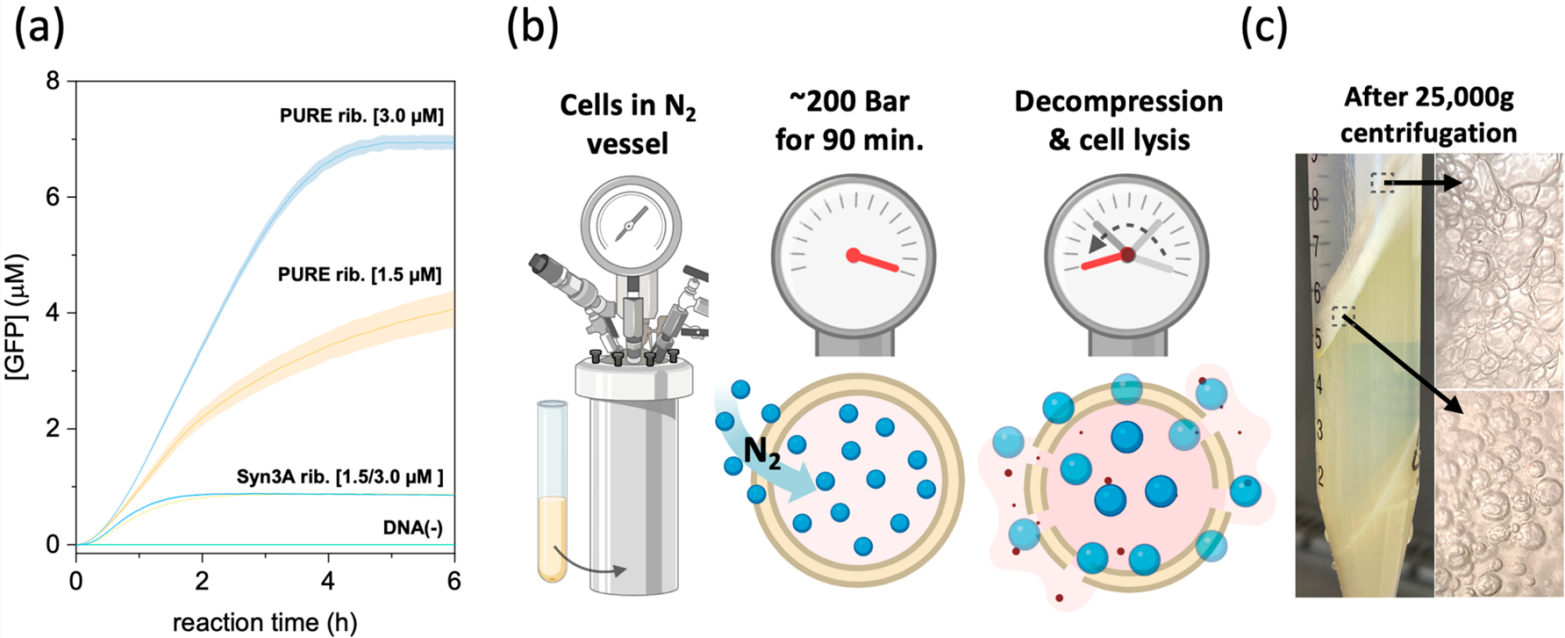
Syn3A ribosome activity and nitrogen decompression method. (a) Expression of GFP protein in PURE CFE platform using the original PURE ribosomes or Syn3A ribosomes. Error bars are standard deviation (*n* = 2). (b) Schematic illustration of the nitrogen decompression method from cell incubation (left), incubation at high nitrogen gas pressure (middle), to cell disruption (right). (c) Foamy layer formed after centrifugation was observed for every crude Syn3A cell lysate prepared (left). Air pockets can be observed under the brightfield microscope (right).

Previously, we reported considerable degradation levels of mRNA (mRNA) and rRNA (rRNA) in *Mycoplasma* lysate, which impeded *in vitro* expression.^11^ In a first breakthrough, we found functional ribosomes in a specific cell lysate derived from Syn3A cells trypsinized before cell disruption (French press) and centrifuged at high speeds (34,000–80,000*g*). Such ribosomes (trypsin-Syn3A ribosomes) were able to express green fluorescent protein (GFP) in a PURE (protein synthesis using recombinant elements) CFE system^17^ (Figure 1a). Despite the lower GFP expression level (9-fold) compared to equimolar ribosome concentration in the PURE reaction, this is (i) the first observation of *in vitro* translation by Syn3A ribosomes and (ii) an indication that RNase activity could be controlled by the lysate preparation method. The lower expression of GFP by Syn3A ribosomes compared to PURE ribosomes could be explained by the lower purity of Syn3A ribosomes (no chromatographic isolation), possible partial degradation (by remaining RNases), or the suboptimal composition of the PURE feeding buffer (optimized for *E. coli*).

**Figure 2:**
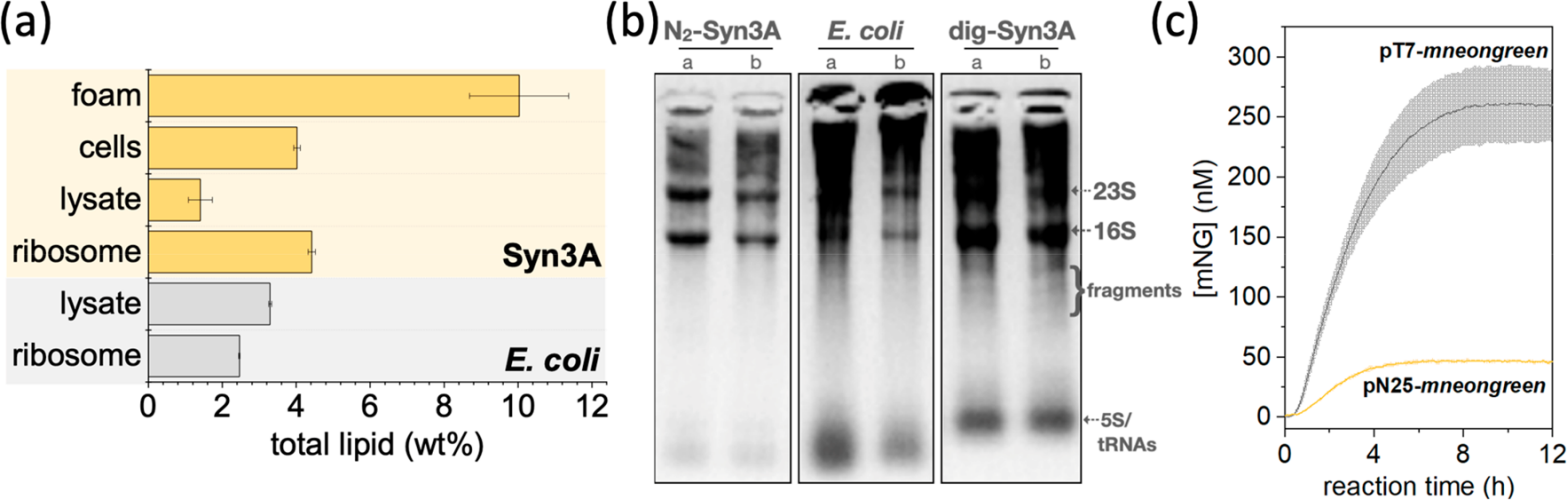
Building a functional Syn3A CFE system. (a) Total lipid analysis of foamy layer compared to other fractions of Syn3A and *E. coli*. Error bars are standard deviation (*n* = 4). (b) Analysis of rRNA (rRNA) stability in N_2_-Syn3A lysate (left box) compared to rRNA from *E. coli* lysate (middle box) and digitonin-derived Syn3A lysate (right box) after incubation at 37 °C for 1h. Right lane is a diluted replicate of the left lane for each box. (c) Expression of mNG by a complete Syn3A CFE system (N_2_-Syn3A lysate and Syn3A ribosomes) controlled by exogenous T7 RNA polymerase (gray line) or endogenous RNA polymerase (blue line). Error bars are standard deviation (*n* = 2). Results are representative of two biological replicates.

Inspired by these early results, we explored other lysis methods aiming for rapid separation of cell membranes and cytoplasm. We, therefore, tested the so-called nitrogen decompression method, which has been used to isolate membrane fractions from *Mycoplasma hominis*(12) and to isolate cytosolic compartments from eukaryotic cells.^13,14^ Syn3A cells were incubated at a high nitrogen gas pressure inside a nitrogen vessel (200 bar, 0 °C, 90 min.) to allow the dissolution of nitrogen gas into cells (Figure 1b). Then, rapid decompression led the dissolved nitrogen gas to expand, provoking cell disruption. Surprisingly, after centrifugation of the crude cell lysate at 25,000*g*, a foamy layer emerged from the supernatant (Figure 1c) and was easily removed. Total lipid analysis revealed a higher concentration of lipids in the foam (10 wt %) compared to that in Syn3A whole cells (4 wt %), Syn3A lysate prepared by nitrogen decompression (N_2_-Syn3A lysate) (1.4 wt %), or Syn3A ribosome solution (4.4 wt %) (Figure 2a). We also found rRNAs to be more stable in N_2_-Syn3A lysate than in digitonin-derived Syn3A lysate (samples incubated at 37 °C for 60 min) (Figure 2b). Encouraged by the production of Syn3A lysates with lower content of membranes and reduced RNase activity, we tested the CFE functionality of N_2_-Syn3A lysates.

To our delight, N_2_-Syn3A lysate was capable of *in vitro* expression of mNeonGreen protein (mNG)^18^ from a plasmid DNA template up to 200–350 nM (5.3–9.3 μg/mL). *In vitro* expression of mNG protein was observed when using either the exogenously added RNA polymerase (T7 RNAP) (Figure 2c, gray) or the endogenous RNAP already present in N_2_-Syn3A lysate (Figure 2c, yellow). The difference in expression levels observed for the Syn3A CFE system containing the exogenous T7 RNAP or endogenous RNAP was likely related to higher transcriptional processivity by T7 RNAP. In both cases, the CFE system required the addition of purified Syn3A ribosomes (Table 1). This could be caused by the intrinsically low density of native ribosomes in Syn3A cells.^19^ With the fully functional Syn3A CFE system (capable of transcription and translation), we focused on optimizing the CFE reaction mixture to improve protein expression.

**Table 1:** Composition of the Syn3A CFE System before and after Optimization for Higher Protein Yield^a^

	Component	Preoptimized composition	Optimized composition
Lysate	Syn3A cell lysate	7.25 mg/mL	5.25 mg/mL
Ribosome	Syn3A ribosome solution	2 μM	2.25 μM
Feeding buffer (FB)	HEPES pH 8.0	50 mM	45 mM
	ATP/GTP/CTP/UTP	2 mM	2 mM
	Amino acid mix	1.5 mM	0.5 mM
	*M*. *capricolum* tRNA	0.2 mg/mL	0.1 mg/mL
	PEG8000	2 wt %	1.5 wt %
	T7 RNA polymerase	150 U/μL	112.5 U/μL
	Ribolock	2.4 U/μL	2 U/μL
	CaCl_2_	15 mM	30 mM
	Mg-glutamate	6 mM	20 mM
	K-glutamate	100 mM	5 mM
	NTP mix	2 mM	2 mM
	3-PGA	30 mM	20 mM
	PEP	30 mM	70 mM
	CoA	0.26 mM	0.15 mM
	NAD^+^	0.33 mM	0.49 mM
	cAMP	0.75 mM	2 mM
	Folinic acid	0.07 mM	0.07 mM
	Spermidine	0.1 mM	0.25 mM
	DTT	0.1 mM	0.15 mM
DNA template	Plasmid DNA	6 nM	6 nM

a The values represent the final concentration of each component in the CFE reaction mixture.

**Figure 3:**
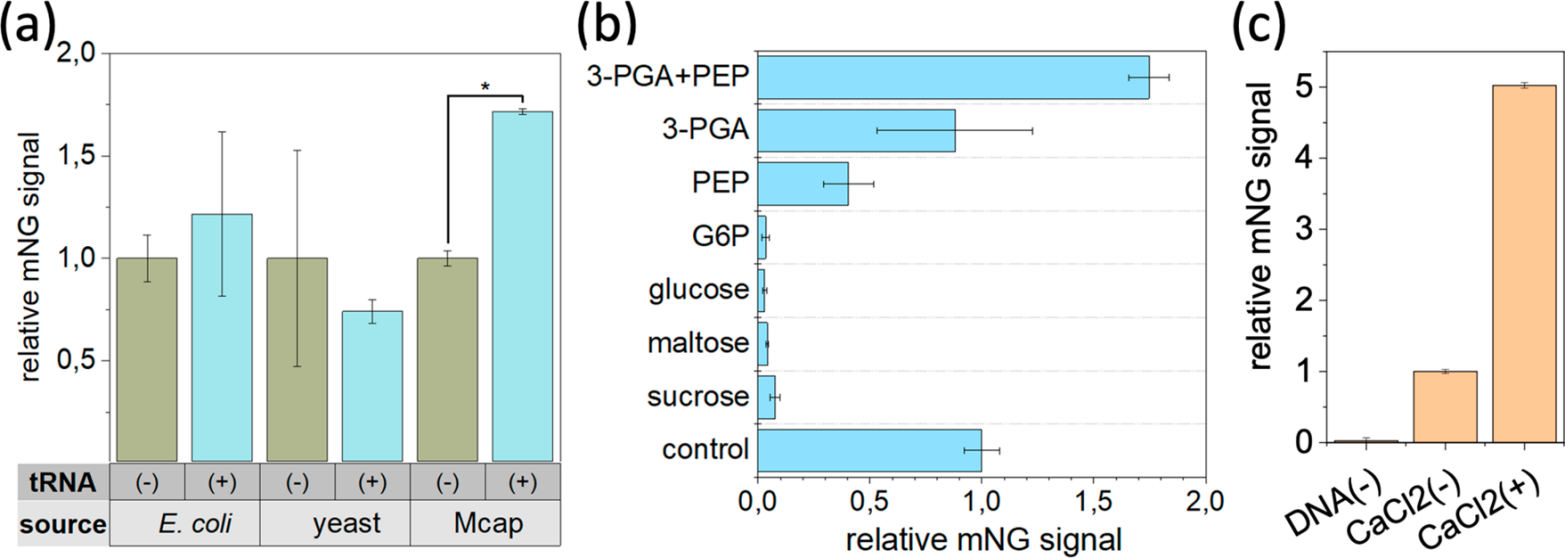
Optimization of feeding buffer components for the Syn3A CFE system. (a) Effect of tRNAs isolated from different organisms in the Syn3A CFE system. Only tRNAs from Mcap show a significant enhancement of Syn3A CFE. (b) Performance of different energy source molecules: 3-PGA, PEP, sucrose, maltose, glucose, and G6P. (c) Effect of CaCl_2_ in the expression of mNG in the Syn3A CFE system. Error bars are relative standard deviation (*n* = 2); *one-way ANOVA, *p* ≤ 0.05.

First, we tested the essentiality of three major components (tRNAs, energy source molecules, and CaCl_2_). Then, a final optimization phase focused on finding the highest protein yielding CFE reaction mixture compositions (a total of 20 components). To assess the importance of tRNAs, we tested the compatibility of tRNAs from (i) nonrelated organisms (*E. coli* and *S. cerevisiae*) and a closely related organism (*Mycoplasma capricolum* subsp. *capri*, Mcap) with the Syn3A CFE system. The addition of tRNAs from *E. coli* or *S. cerevisiae* tRNAs had a neutral effect in mNG expression (Figure 3a, gray/yellow) while tRNAs from Mcap improved *in vitro* expression (Figure 3a, blue). Next, we tested common energy source molecules for CFE systems: 3-phosphoglyceric acid (3-PGA), phosphoenolpyruvate (PEP), sucrose, maltose, glucose, and glucose-6-phosphate (G6P).^1,20^ As expected, 3-PGA and PEP performed better, and an equimolar combination of them showed the best expression levels (Figure 3b). The last component tested was CaCl_2_, which is a nonconventional element for CFE systems but was previously found as an inhibitor of Mcap nucleases.^11,21^ Indeed, our experiments showed that 15–25 mM CaCl_2_ improved mNG expression (Figure 3c). Therefore, CaCl_2_, 3-PGA, PEP, and Mcap tRNA were included as permanent components of the Syn3A CFE system (Table 1).

**Figure 4:**
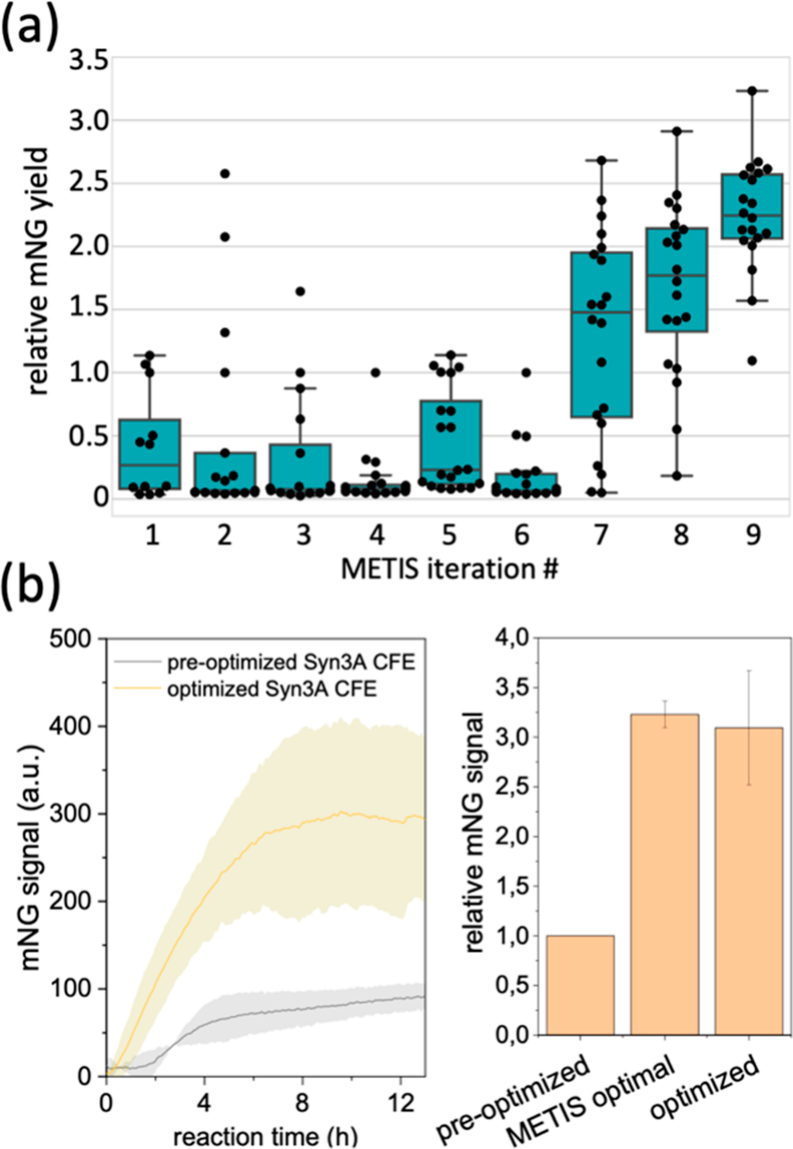
Optimization of the entire reaction mixture for the Syn3A CFE system. (a) Boxplot of normalized protein yield progression per METIS iteration. Each dot is an independent experiment containing a distinct reaction mix composition suggested by METIS. Relative yield is a ratio of (mNG signal for each new reaction mix)/(mNG signal for the preoptimized reaction mix). (b) Expression of mNG using the optimized CFE reaction mix in the complete Syn3A CFE system (N_2_-Syn3A lysate and Syn3A ribosomes). Error bars are standard deviation or relative standard deviation (*n* = 2).

To find the best compositions for the Syn3A CFE reaction mixture, we utilized an active machine learning method using the XGBoost algorithm.^22^ This computational method has been previously adapted to the study of *E. coli*-based CFE systems and was renamed as METIS (machine learning-guided experimental trials for improvement of systems).^15,16^ METIS suggests experimentally testable compositions for the reaction mix, and the results are then used as a training data set to generate a new set of compositions to be tested in the following iteration. Here, we sampled a 20-dimensional space of components for the reaction mix (4^20^ potential combinations) across nine rounds of METIS iterations (15–20 different reaction mix compositions tested per iteration) (Figure 4). At the start of the optimization cycle, conditions are randomly sampled in a global search (exploration). As more data is collected and the model gains predictive power, the search becomes increasingly focused, eventually converging to a local maximum (exploitation).^16^ This process works best for systems that are not subject to large extrinsic perturbations, and the model can map onto a robust, generalizable structure. Because it is known that there is variance between and within lysate batches,^23^ we performed a parallel analysis and found that multilayer perceptron (MLP) models, on average, gained a measure of predictive power after six iterations but noise was still a factor (Figure S1a,b). To account for this, we opted for streamlining the optimization, allowing the algorithm to alter the hitherto five most sensitive variables.

In practice, we prepared CFE reaction mixtures from the stock solution for each component. The first round of METIS is usually performed without any input data or random data. In our case, the first METIS round included the mNG yields obtained for 20 CFE reaction mixtures containing either the maximum or minimum concentration for a single component, while maintaining the other components at the standard concentration (Table 1, preoptimized composition). We also included mNG yields for another 48 random CFE reaction mixtures. Therefore, the first training data set comprised 68 different CFE reaction mixtures. From the second iteration, we tested the CFE reaction compositions suggested by METIS. Until the sixth iteration, we observed no significant improvement in the yields of mNG production, which was probably caused by the large number of variables employed (20 components) and the relatively low number of conditions tested per round (15–20 distinct compositions). METIS studies reported in the literature had up to 11 different components in the CFE reaction mix and they tested 10–100 different conditions per iteration. Therefore, from the seventh iteration, we fixed the concentration of 15 components that presented no variation in the previous iterations. After an additional 3 iterations, we achieved a 3.2-fold increase in mNG protein yield (Figure 4a) compared to the yield obtained with the preoptimized reaction mix (Table 1). The CFE reactions for the METIS optimization used *E. coli* ribosomes due to the limited availability of Syn3A ribosomes. Finally, we demonstrated the robustness of the optimized reaction mix composition with the complete Syn3A CFE system (Syn3A lysate with Syn3A ribosomes) showing a similar increase in expression yield (Figure 4b).

In summary, we have demonstrated that a functional Syn3A CFE system can be prepared and protein production can be measured. The cell disruption method used in this study is unusual for preparing lysates from other prokaryotic organisms due to the unique cellular structure of Syn3A and *Mollicutes* in general (absent cell wall). Compared to traditional cell disruption methods that employ shear forces, nitrogen decompression promotes cell lysis from the inside out during nitrogen gas expansion. The formation of gas from inside the cells might have allowed the formation of the lipid-rich foam layer observed after the centrifugation of the crude lysate. In addition to membrane isolation by centrifugal forces, nitrogen decompression allowed further removal of membrane fragments through foaming. Better removal of membranes is potentially the main reason for the reduced RNase activity observed for N_2_-Syn3A lysates.

Compared to CFE systems derived from other organisms, the Syn3A CFE platform yields the lowest concentrations of protein.^20,24^ For instance, *E. coli* and *Streptomyces venezuelae* CFE systems can produce up to 2500 μg/mL and 266 μg/mL of protein, respectively. This means up to a 280-fold difference in protein yield compared to that for the Syn3A CFE system (350 nM, 9.3 μg/mL). Considering that the *E. coli* CFE system has been developed for more than six decades,^25,26^ we recognize that the Syn3A CFE system still needs further optimization to improve CFE yields and robustness.

For example, we noticed a high variability in the functionality of ribosomes isolated from Syn3A cells. The ribosome batch that yielded the highest mNG production was isolated from Syn3A cells that were trypsinized before cell disruption (Figure 2b). Depending on the ribosome batch, the mNG yield varied by 1 order of magnitude. Possibly, ribosome isolates purified from N_2_-Syn3A cell lysates have a higher concentration of membrane-associated nucleases than in the lysate due to ultracentrifugation (173,000*g*). This hypothesis is supported by the higher concentration of lipids (from membrane fragments) in the ribosome isolates (Figure 2a). For this study, the batch-to-batch variation problem was circumvented by using a single and large batch of Syn3A lysate and Syn3A ribosomes. For the METIS experiments, we employed ribosomes isolated from *E. coli*, which presented significantly lower variation between different batches. The functionality of Syn3A ribosome isolates can be possibly improved by (i) controlling the Syn3A cell harvesting time (at mid log instead of stationary phase), (ii) improving the removal of membrane-associated nucleases of Syn3A before cell disruption, (iii) using Ni-NTA Sepharose for the isolation of hexahistidine-tagged ribosomes to avoid the use of ultracentrifugation, or (iv) employing ribosomes with modified rRNAs for improved stability.

Although improvement in protein yields will certainly be the focus of further studies, we also realize that other features are important for the purpose of building a synthetic cell. For example, the minimal synthetic cell will need to express several genes instead of a single protein in high yields. Therefore, efforts to improve the Syn3A system should also be directed towards developing tools to control multiplex gene expression (e.g., characterization of genetic regulators, synthetic genomics).

In conclusion, we have demonstrated the first functional *Mycoplasma*-based CFE system. This is a major breakthrough for the bottom-up construction of a minimal synthetic cell. By using Syn3A, which contains only 493 genes, as a donor organism, in contrast to commonly used *E. coli* (4,401 genes), the level of complexity encountered in reconstructing the full functionality of the living cell has decreased dramatically.

## Materials and Methods

### Chemicals and Reagents

RiboLock RNase inhibitor (EO0382) and dithiothreitol (DTT) (10386833) were purchased from Thermo Fisher Scientific (USA). HEPES (2-[4-(2-hydroxyethyl)-1-piperazinyl]ethanesulfonic acid) (13957028) was purchased from VWR Chemicals (USA). Adenosine 3′,5′-cyclic monophosphate (cAMP) (A9501), calcium chloride dihydrate (CaCl_2_) (2382), coenzyme A hydrate (coA) (C4282), l-glutamic acid, hemimagnesium salt tetrahydrate (Mg-glut) (49605), β-nicotinamide adenine dinucleotide 94% (NAD^+^) (n8881), poly(ethylene glycol) Mw ∼ 8,000 (PEG8000) (202452), and spermidine (s0266) were purchased from Sigma-Aldrich (USA). Adenosine 5′-triphosphate, disodium salt trihydrate (ATP) (NU-1010), guanosine 5′-triphosphate, disodium salt trihydrate (GTP) (NU-1047), cytidine 5′-triphosphate, disodium salt trihydrate (CTP) (NU-1047), uridine 5′-triphosphate, and trisodium salt trihydrate (UTP) (NU-1013) were purchased from Jena Bioscience (Germany). Folinic acid, calcium salt (sc-252837A), and d-(−)-3-phosphoglyceric acid and disodium salt (3-PGA) (sc-214793B) were purchased from Santa Cruz Biotechnology (USA). Phosphoenolpyruvic acid, monopotassium salt (PEP) (B20358), and l-glutamic acid, potassium salt (K-glut) (A17232.0B) were purchased from Alfa Aesar (USA). Chemicals or reagents for a particular protocol are mentioned in the respective following sections.

### Micro-organisms and Culturing Conditions

We used the JCVI-syn3A strain (Syn3A, GenBank: CP016816.2) to produce the cell lysate and to isolate ribosomes.^4^ Hayflick medium:^27^ 21 g/L of *Mycoplasma* broth base (CM0403B, Oxoid, Thermo Fisher Scientific, USA), 5 g/L dextrose (D9559, Sigma-Aldrich, USA), 15 g/L yeast extract (16229771, Gibco, Thermo Fisher Scientific, USA), 20 vol % of heat-inactivated horse serum (26050088, Gibco, Thermo Fisher Scientific, USA), and 400,000 U/L of penicillin sodium salt (P3032, Sigma-Aldrich, USA). Cells were cultured at 37 °C with mild agitation (120 rpm) until the stationary phase (pH 6.5). The doubling time of Syn3A cells is about 2 h, and the cell growth curve can be found in our previous study.^4^ The pH indicator phenol red dye was omitted from the cultures to avoid contamination of the final lysate. Syn3A cells were harvested at the stationary growth phase (∼pH 6.5) for enhanced biomass levels. The culture media was centrifuged (10,000*g*, 15 min, 4 °C), and the cell pellets by were washed with the HEPES buffer (75 mM, pH 8) or the S30A buffer (50 mM Tris pH 7.7, 14 mM Mg-glutamate, 60 mM K-glutamate, pH 7.6).^28^ The washed pellets were used for lysate preparation (frozen pellets yielded lysates with significantly lower functionality in CFE reaction) and ribosome isolation. *E. coli* was cultured as described by Sun et al.^28^*E. coli* lysate was also used for ribosome isolation. *Mycoplasma capricolum* subsp. *capricolum* (GenBank: CP000123.1) cells were cultured similarly to Syn3A except for agitation (125 rpm).

### Plasmid DNA Templates

The plasmid DNA containing the *mneongreen* gene controlled by pN25 promoter (pMflT-04-*mneongreen*) was a gift from Prof. Sébastien Rodrigue (Addgene plasmid #201855). From the pMflT-o4-*mneongreen* plasmid, the *mneongreen* gene was designed under the control of the T7 promoter and cloned into the pUCIDT vector (Amp^R^) (Integrated DNA Technologies, USA). The final plasmid was sequence verified and transformed into *E. coli* Top10 chemically competent cells (C404006, Invitrogen, USA) for storage and amplification.

### Syn3A Cell Lysate Preparation by Nitrogen Decompression

This protocol was adapted from several literature references.^12−14,29^ The cell pellets were washed twice with the S30A buffer (50 mM Tris pH 8.2, 14 mM Mg-glutamate, 60 mM K-glutamate) and resuspended in the S30B buffer (5 mM Tris pH 8.2, 14 mM Mg-glutamate, 60 mM K-glutamate) using 1 mL of S30B per gram of pellet. The cell suspension was transferred to the nitrogen vessel (Series 4639/T304SS, Parr Instrument Corporation, USA) and pressurized at 200 bar on ice for 90 min. A magnetic stirring bar was used to keep the cell suspension homogeneous during incubation. The high pressure was released by slowly opening the lower outlet channel, through which the crude lysate is drained into a precooled 15 mL centrifuge tube. After centrifugation at 25,000*g* (12 min, 4 °C), the floating foamy layer (formed in Syn3A) was removed with a spatula and the supernatant was collected. The lysate was then aliquoted in fractions of 500 μL and incubated at 30 °C (80 min, 350 rpm, open lid) to release ribosomes from mRNAs (runoff incubation).^30^ A second centrifugation step (25,000*g*, 12 min, 4 °C) followed the runoff incubation. The lysate was then dialyzed against the S30B buffer supplemented with 1 mM DTT for 3 h at 4 °C using a MWCO 3500 Da dialysis membrane (Spectra Por 7, Repligen Corporation, USA). A final centrifugation step (25,000*g*, 12 min, 4 °C) was carried out before aliquoting, flash freezing, and storing at −80 °C. The protein concentration of the final lysate was around 21 mg/mL (batch specific).

### Rough Ribosome Fraction Isolation

This protocol was adapted from literature references.^31,32^ Ribosomes were isolated from previously prepared cell lysates. To better remove cellular debris, the cell lysate was centrifuged at 50,000*g* (15 min, 4 °C) before runoff incubation and at 34,000*g* (15 min, 4 °C) afterward. Usually, dialysis was skipped for lysate preparations that were used for the isolation of ribosomes. The lysate was then diluted into the S30B buffer containing 1 mM DTT (1:2; lysate:S30B), loaded into ultracentrifugation tubes, (10.8 mL tube, Ti-90 rotor, Beckman-Coulter, USA), and centrifuged at 173,500*g* for 3 h, 4 °C. The supernatant was discarded, and the pellet was rinsed once with the S30B buffer. The pellets were then resuspended in S30B supplemented with 1 mM DTT (Sigma-Aldrich, USA), aliquoted, and stored at −80 °C. For measuring ribosome concentration, samples were 1000× diluted in deionized water, and the absorbance was measured at 260 nm (A260, Nanodrop, Thermo Fisher Scientific, USA). The A260 signal was converted to ribosome concentration using a calibration curve prepared with standard ribosomes from a commercial CFE kit (PUREfrex2.0, GeneFrontier, Japan).

### Isolation of Mcap tRNA

Bulk tRNA from *Mycoplasma capricolum* subsp. *capri* was isolated according to the method described by Avcilar-Kucukgoze et al.^33^ with minor alterations. *Mycoplasma capricolum* subsp. *capri* cell pellets from 500 mL of culture were washed once with Tris-Sucrose Buffer (0.5 M sucrose, 10 mM Tris pH 6.5) and centrifuged at 10,000*g* for 20 min at 4 °C. The supernatant was discarded, and the cells were resuspended in 18 mL of a nucleic acid extraction buffer (50 mM sodium acetate (NaOAc), and 10 mM magnesium acetate, pH 5.0). Total nucleic acids were extracted by adding 17.2 mL of acidic phenol pH 4.5 (VWR, USA) to the resuspended cells and were shaken at 215 rpm for 30 min at 20 °C. The cell emulsion was centrifuged at 5,000*g* for 15 min at 4 °C and the aqueous phase was collected. The emulsion was extracted again as above by adding 14 mL of nucleic acid extraction buffer to the slurry and repeating both shaking and centrifugation steps. Following a second centrifugation, the aqueous phase was collected and pooled with the aqueous phase from the first extraction. Total nucleic acids were precipitated from the collected aqueous phases above by adding 1.5 mL of 5 M NaCl and one volume of isopropyl alcohol followed by centrifugation at 14,500*g* at 20 °C for 15 min. Precipitated nucleic acids were washed once with 70% ethanol and allowed to dry. Ribosomal RNAs were precipitated from the total nucleic acid fraction by resuspending the nucleic acid pellet in 15 mL of cold 1 M NaCl followed by centrifugation at 9,500*g* for 20 min at 4 °C. The remaining nucleic acids were precipitated from the collected supernatant by adding 30 mL of ice-cold ethanol. This solution was allowed to incubate for 30 min at −20 °C before being centrifuged at 14,500*g* for 5 min at 20 °C. The resulting pellet was washed once with 70% ethanol and allowed to air-dry. The pellet was resuspended in 6 mL of 0.3 M NaOAc pH 5.0 followed by the addition of 3.4 mL of isopropyl alcohol. The solution was allowed to incubate for 10 min at 20 °C before it was centrifuged at 14,500*g* for 5 min at 20 °C. The supernatant was collected, and 2.3 mL of isopropyl alcohol was added. The solution was allowed to precipitate overnight at −20 °C before centrifugation at 14,500*g* for 15 min at 4 °C. The tRNA pellet was washed once with 70% ethanol and allowed to air-dry before resuspension in 250 μL of RNase-free H_2_O. The concentration of the tRNA solution was quantitated by measuring A260 (Nanodrop, Thermo Fisher Scientific, USA).

### Cell-Free Expression Reaction

The reaction mixture also contained Syn3A cell lysate and ribosomes (described above), tRNAs isolated from *Mycoplasma capricolum* subsp. *capri* cells (Mcap tRNA), and the plasmid DNA template (described above). The CFE reaction was performed in 384-well plates (black, flat-bottom, 781900, Greiner Bio-one, Austria) using 11 μL sample/well and incubated at 30 °C. Fluorescence was measured using filters for excitation (485 nm) and emission (520 nm) every 5 min for 24 h (Spark M10 Spark, Tecan, Austria).

### Active Machine Learning (METIS) Pipeline

The original METIS and user guides are available online and open-access at GitHub and can be used in the Google Colab platform.^16^ In this study, the “METIS_optimization_Notebook” program was used. Protein yields were shown as a ratio between the mNG fluorescence signal for each new reaction mix composition divided by the fluorescence signal of the standard sample (containing the preoptimized reaction mix). Each new reaction mix was measured in duplicates.

### Data Analysis and Presentation

Data was analyzed on Microsoft Excel, graphs were produced with Origin 8, and illustrations were created in BioRender.

## Supporting Information Available

Figure S1 (METIS predictive power and MLP) (PDF)

## Abbreviations

(CFE): Cell-free expression
(Syn3A): JCVI-syn3A
(Mcap): *Mycoplasma capricolum* subsp. *capri*
(*E. coli*): *Escherichia coli*
(*S. cerevisiae*): *Saccharomyces cerevisiae*
(RNase): ribonuclease
(PURE): protein synthesis using recombinant elements
(GFP): green fluorescent protein
(mNG): mNeonGreen protein
(mRNA): messenger ribonucleic acid
(rRNA): ribosomal ribonucleic acid
(tRNA): transfer ribonucleic acid
(DNA): DNA
(N_2_): nitrogen gas
(g): gravitational force
(dig): digitonin
(RNAP): RNA polymerase
(CaCl_2_): calcium chloride
(3-PGA): 3-phosphoglyceric acid
(PEP): phosphoenolpyruvate
(G6P): glucose-6-phosphate
METIS: (machine learning-guided experimental trials for improvement of systems)
(R^2^): coefficient of determination
(Amp^R^): ampicillin resistant
(RPM): rotations per minute

## References

[ref1] Garenne D.; Thompson S.; Brisson A.; Khakimzhan A.; Noireaux V. The All-E. ColiTXTL Toolbox 3.0: New Capabilities of a Cell-Free Synthetic Biology Platform. Synth Biol. (Oxf) 2021, 6 (1), ysab017 10.1093/synbio/ysab017. 34712841 PMC8546610

[ref2] Gibson D. G.; Glass J. I.; Lartigue C.; Noskov V. N.; Chuang R.-Y.; Algire M. A.; Benders G. A.; Montague M. G.; Ma L.; Moodie M. M.; Merryman C.; Vashee S.; Krishnakumar R.; Assad-Garcia N.; Andrews-Pfannkoch C.; Denisova E. A.; Young L.; Qi Z.-Q.; Segall-Shapiro T. H.; Calvey C. H.; Parmar P. P.; Hutchison C. A.; Smith H. O.; Venter J. C. Creation of a Bacterial Cell Controlled by a Chemically Synthesized Genome. Science 2010, 329 (5987), 52–56. 10.1126/science.1190719. 20488990

[ref3] Hutchison C. A.; Chuang R.-Y.; Noskov V. N.; Assad-Garcia N.; Deerinck T. J.; Ellisman M. H.; Gill J.; Kannan K.; Karas B. J.; Ma L.; Pelletier J. F.; Qi Z.-Q.; Richter R. A.; Strychalski E. A.; Sun L.; Suzuki Y.; Tsvetanova B.; Wise K. S.; Smith H. O.; Glass J. I.; Merryman C.; Gibson D. G.; Venter J. C. Design and Synthesis of a Minimal Bacterial Genome. Science 2016, 351 (6280), aad6253 10.1126/science.aad6253. 27013737

[ref4] Breuer M.; Earnest T. M.; Merryman C.; Wise K. S.; Sun L.; Lynott M. R.; Hutchison C. A.; Smith H. O.; Lapek J. D.; Gonzalez D. J.; de Crécy-Lagard V.; Haas D.; Hanson A. D.; Labhsetwar P.; Glass J. I.; Luthey-Schulten Z. Essential Metabolism for a Minimal Cell. Elife 2019, 8. 10.7554/eLife.36842. PMC6609329 30657448

[ref5] Bianchi D. M.; Pelletier J. F.; Hutchison C. A.; Glass J. I.; Luthey-Schulten Z. Toward the Complete Functional Characterization of a Minimal Bacterial Proteome. J. Phys. Chem. B 2022, 126 (36), 6820–6834. 10.1021/acs.jpcb.2c04188. 36048731 PMC9483919

[ref6] Thornburg Z. R.; Melo M. C. R.; Bianchi D.; Brier T. A.; Crotty C.; Breuer M.; Smith H. O.; Hutchison C. A.; Glass J. I.; Luthey-Schulten Z. Kinetic Modeling of the Genetic Information Processes in a Minimal Cell. Front Mol. Biosci 2019, 6, 130 10.3389/fmolb.2019.00130. 31850364 PMC6892953

[ref7] Yamao F.; Muto A.; Kawauchi Y.; Iwami M.; Iwagami S.; Azumi Y.; Osawa S. UGA Is Read as Tryptophan in Mycoplasma Capricolum. Proc. Natl. Acad. Sci. U. S. A. 1985, 82 (8), 2306–2309. 10.1073/pnas.82.8.2306. 3887399 PMC397546

[ref8] Fujiwara K.; Sawamura T.; Niwa T.; Deyama T.; Nomura S. I. M.; Taguchi H.; Doi N. In Vitro Transcription-Translation Using Bacterial Genome as a Template to Reconstitute Intracellular Profile. Nucleic Acids Res. 2017, 45 (19), 11449–11458. 10.1093/nar/gkx776. 28977538 PMC5737407

[ref9] Lartigue C.; Glass J. I.; Alperovich N.; Pieper R.; Parmar P. P.; Hutchison C. A.; Smith H. O.; Venter J. C. Genome Transplantation in Bacteria: Changing One Species to Another. Science 2007, 317 (5838), 632–638. 10.1126/science.1144622. 17600181

[ref10] Baby V.; Labroussaa F.; Brodeur J.; Matteau D.; Gourgues G.; Lartigue C.; Rodrigue S. Cloning and Transplantation of the Mesoplasma Florum Genome. ACS Synth. Biol. 2018, 7 (1), 209–217. 10.1021/acssynbio.7b00279. 28893065

[ref11] Sakai A.; Deich C. R.; Nelissen F. H. T.; Jonker A. J.; Bittencourt D. M. de C.; Kempes C. P.; Wise K. S.; Heus H. A.; Huck W. T. S.; Adamala K. P.; Glass J. I. Traditional Protocols and Optimization Methods Lead to Absent Expression in a Mycoplasma Cell-Free Gene Expression Platform. Synth Biol. (Oxf) 2022, 7 (1), ysac008 10.1093/synbio/ysac008. 35774105 PMC9239315

[ref12] Hollingdale M. R.; Lemcke R. M. The Antigens of Mycoplasma Hominis. J. Hyg (Lond) 1969, 67 (4), 585–602. 10.1017/S0022172400042042. 4982556 PMC2130762

[ref13] Simpson R. J. Disruption of Cultured Cells by Nitrogen Cavitation. Cold Spring Harb Protoc 2010, 2010 (11), pdb.prot5513 10.1101/pdb.prot5513. 21041386

[ref14] Zhou M.; Philips M. R. Nitrogen Cavitation and Differential Centrifugation Allows for Monitoring the Distribution of Peripheral Membrane Proteins in Cultured Cells. J. Vis Exp 2017, 2017 (126), 56037 10.3791/56037. PMC5614342 28872138

[ref15] Borkowski O.; Koch M.; Zettor A.; Pandi A.; Batista A. C.; Soudier P.; Faulon J. L. Large Scale Active-Learning-Guided Exploration for in Vitro Protein Production Optimization. Nat. Commun. 2020, 11 (1), 1–8. 10.1038/s41467-020-15798-5. 32312991 PMC7170859

[ref16] Pandi A.; Diehl C.; Yazdizadeh Kharrazi A.; Scholz S. A.; Bobkova E.; Faure L.; Nattermann M.; Adam D.; Chapin N.; Foroughijabbari Y.; Moritz C.; Paczia N.; Cortina N. S.; Faulon J.-L.; Erb T. J. A Versatile Active Learning Workflow for Optimization of Genetic and Metabolic Networks. Nat. Commun. 2022, 13 (1), 3876 10.1038/s41467-022-31245-z. 35790733 PMC9256728

[ref17] Shimizu Y.; Inoue A.; Tomari Y.; Suzuki T.; Yokogawa T.; Nishikawa K.; Ueda T. Cell-Free Translation Reconstituted with Purified Components. Nat. Biotechnol. 2001, 19 (8), 751–755. 10.1038/90802. 11479568

[ref18] Shaner N. C.; Lambert G. G.; Chammas A.; Ni Y.; Cranfill P. J.; Baird M. A.; Sell B. R.; Allen J. R.; Day R. N.; Israelsson M.; Davidson M. W.; Wang J. A Bright Monomeric Green Fluorescent Protein Derived from Branchiostoma Lanceolatum. Nat. Methods 2013, 10 (5), 407–409. 10.1038/nmeth.2413. 23524392 PMC3811051

[ref19] Gilbert B. R.; Thornburg Z. R.; Lam V.; Rashid F.-Z. M.; Glass J. I.; Villa E.; Dame R. T.; Luthey-Schulten Z. Generating Chromosome Geometries in a Minimal Cell From Cryo-Electron Tomograms and Chromosome Conformation Capture Maps. Front Mol. Biosci 2021, 8, 644133 10.3389/fmolb.2021.644133. 34368224 PMC8339304

[ref20] Moore S. J.; Lai H.-E.; Chee S.-M.; Toh M.; Coode S.; Chengan K.; Capel P.; Corre C.; de Los Santos E. L.; Freemont P. S. A Streptomyces Venezuelae Cell-Free Toolkit for Synthetic Biology. ACS Synth. Biol. 2021, 10 (2), 402–411. 10.1021/acssynbio.0c00581. 33497199 PMC7901020

[ref21] Minion F. C.; Jarvill-Taylor K. J.; Billings D. E.; Tigges E. Membrane-Associated Nuclease Activities in Mycoplasmas. J. Bacteriol. 1993, 175 (24), 7842–7847. 10.1128/jb.175.24.7842-7847.1993. 8253673 PMC206960

[ref22] Chen T.; Guestrin C. XGBoost: A Scalable Tree Boosting System. In Proceedings of the 22nd ACM SIGKDD International Conference on Knowledge Discovery and Data Mining, August 13–17, 2016, San Francisco CA; ACM: New York, NY, USA, 2016; pp 785–794. 10.1145/2939672.2939785.

[ref23] Dopp J. L.; Jo Y. R.; Reuel N. F. Methods to Reduce Variability in E. Coli-Based Cell-Free Protein Expression Experiments. Synth Syst. Biotechnol 2019, 4 (4), 204–211. 10.1016/j.synbio.2019.10.003. 31750411 PMC6849339

[ref24] Gregorio N. E.; Levine M. Z.; Oza J. P. A User’s Guide to Cell-Free Protein Synthesis. Methods Protoc 2019, 2 (1), 24 10.3390/mps2010024. 31164605 PMC6481089

[ref25] Nirenberg M. W.; Matthaei J. H. The Dependence of Cell-Free Protein Synthesis in E. Coli upon Naturally Occurring or Synthetic Polyribonucleotides. Proc. Natl. Acad. Sci. U. S. A. 1961, 47 (10), 1588–1602. 10.1073/pnas.47.10.1588. 14479932 PMC223178

[ref26] Nirenberg M. W. [3] Cell-Free Protein Synthesis Directed by Messenger RNA. In Methods in Enzymology; Academic Press, 1963; Vol. 6, pp 17–23. 10.1016/0076-6879(63)06142-5.

[ref27] Wise K. S.; Watson R. K. Mycoplasma Hyorhinis GDL Surface Protein Antigen P120 Defined by Monoclonal Antibody. Infect. Immun. 1983, 41 (3), 1332–1339. 10.1128/iai.41.3.1332-1339.1983. 6604027 PMC264643

[ref28] Sun Z. Z.; Hayes C. A.; Shin J.; Caschera F.; Murray R. M.; Noireaux V. Protocols for Implementing an Escherichia Coli Based TX-TL Cell-Free Expression System for Synthetic Biology. J. Vis Exp 2013, 79, e50762 10.3791/50762. PMC3960857 24084388

[ref29] Foucher A. L.; Papadopoulou B.; Ouellette M. Prefractionation by Digitonin Extraction Increases Representation of the Cytosolic and Intracellular Proteome of Leishmania Infantum. J. Proteome Res. 2006, 5 (7), 1741–1750. 10.1021/pr060081j. 16823982

[ref30] Subeamanian A. R.; Davis B. D. Release of 70 S Ribosomes from Polysomes in Escherichia Coli. J. Mol. Biol. 1973, 74 (1), 45–56. 10.1016/0022-2836(73)90353-7. 4581287

[ref31] Lavickova B.; Maerkl S. J. A Simple, Robust, and Low-Cost Method To Produce the PURE Cell-Free System. ACS Synth. Biol. 2019, 8 (2), 455–462. 10.1021/acssynbio.8b00427. 30632751

[ref32] Grasemann L.; Lavickova B.; Elizondo-Cantú M. C.; Maerkl S. J. OnePot PURE Cell-Free System. J. Vis Exp 2021, 2021 ( (172), ). 10.3791/62625. 34251370

[ref33] Avcilar-Kucukgoze I.; Gamper H.; Hou Y. M.; Kashina A. Purification and Use of TRNA for Enzymatic Post-Translational Addition of Amino Acids to Proteins. STAR Protoc 2020, 1 (3), 100207 10.1016/j.xpro.2020.100207. 33377101 PMC7757669

