# Supplementary material for: Cell-Free Expression System Derived from a Near-Minimal Synthetic Bacterium
Source: ACS Synth Biol. 2023 Jun 6;12(6):1616–23. doi: 10.1021/acssynbio.3c00114 (PMC10278164; doi:10.1021/acssynbio.3c00114)
Supplement: Supplementary file 1 — sb3c00114_si_001.pdf [file sb3c00114_si_001.pdf]

Supplementary Information

A cell-free expression system derived from a near-minimal synthetic bacterium.

Andrei Sakai[1], Aafke J. Jonker[1], Frank H. T. Nelissen[1], Evan M. Kalb[2], Bob van Sluijs[1], Hans A. Heus[1], Katarzyna P. Adamala[2], John I. Glass[3], Wilhelm T. S. Huck[1]*.

[1] Institute for Molecules and Materials, Radboud University, Nijmegen, 6525AJ, The Netherlands
[2] Department of Genetics, Cell Biology and Development, University of Minnesota, Minneapolis, Minnesota 55455, USA
[3] Synthetic Biology & Bioenergy, J. Craig Venter Institute, La Jolla, California 92037, USA
*Corresponding author: w.huck@science.ru.nl

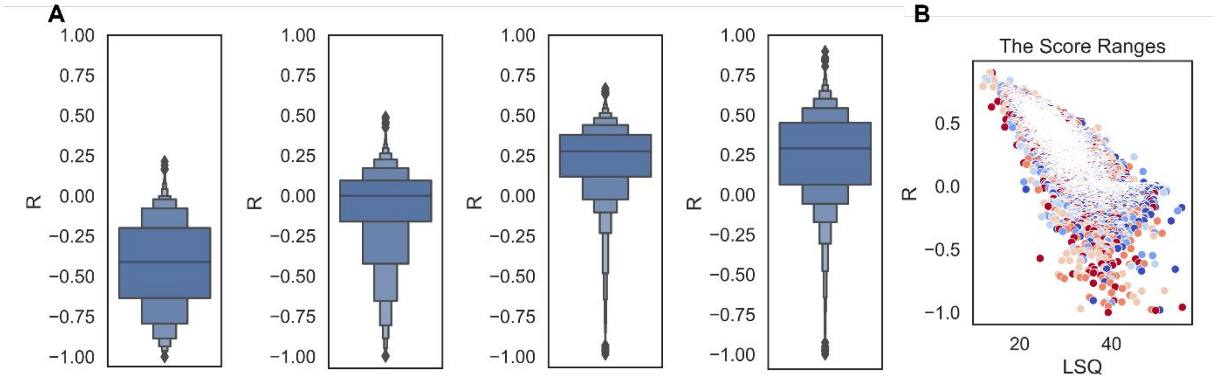

Figure S1. (a) Increase in the predictive power of models as a larger data fraction is added to the training dataset, from left to right, 10%, 30%, 70%, 90% of the whole dataset is used as training data. (b) Individual models (each dot) were trained on 90% of the data and the colours represent the number of hidden layers in the multilayer perceptron (MLP, 2-7 from blue to red).
